# Woven Carbon-Fiber-Reinforced Polymer Tubular Mesh Reinforcement of Hollow High-Performance Concrete Beams

**DOI:** 10.3390/polym15143089

**Published:** 2023-07-19

**Authors:** Jakub Řepka, Tomáš Vlach, Jakub Hájek, Richard Fürst, Jan Pošta, Petr Hájek

**Affiliations:** 1 University Centre for Energy Efficient Buildings, Czech Technical University in Prague, 27343 Buštěhrad, Czech Republic; tomas.vlach@cvut.cz (T.V.); jakub.hajek@cvut.cz (J.H.); jan.posta@cvut.cz (J.P.); petr.hajek@fsv.cvut.cz (P.H.); 2 Federal Institute for Materials Research and Testing (BAM), Division 7.3-Fire Engineering, Unter den Eichen 87, 12205 Berlin, Germany; richard.fuerst@bam.de

**Keywords:** carbon-fiber-reinforced polymer, woven composite reinforcement, hollow concrete beams, high-performance concrete, bond strength, sand-coated, PVA fibers

## Abstract

This article presents woven carbon-fiber-reinforced polymer (CFRP) tubular mesh used as a reinforcement on the inner surface of hollow beams made of high-performance concrete (HPC). The tubular mesh was designed to serve as both the tensile and shear reinforcement of hollow beams intended for the construction of small self-supporting structures that could be assembled without mechanization. The reinforcement was prepared with a tri-axial weaving machine from carbon filament yarn and was homogenized using epoxy resin. The interaction of the composite reinforcement with the cementitious matrix was investigated, and the surface of the reinforcement was modified using silica sand and polyvinyl alcohol (PVA) fibers to improve cohesion. The sand coating enhanced bond strength, resulting in the significantly higher flexural strength of the hollow beam of 128%. The PVA fibers had a lower positive effect of 64% on the flexural strength but improved the ductility of the beam. Individual beams were connected by gluing steel parts directly inside the hollow core of the HPC beam. This procedure provides good interaction between the CFRP reinforcement and the glued steel insert and allows for the fast and simple assembly of structures. The weaving of additional layers of the CFRP reinforcement around HPC beams was also explored. A small structure made of the hollow HPC beams with inner composite reinforcement was constructed to demonstrate the possibilities of the presented technology.

## 1. Introduction

High-performance concrete (HPC) is widely used not only for its good mechanical properties but also for its aesthetic qualities. Combined with composite reinforcement, HPC can be used in a wide range of applications while being significantly more weight efficient compared to traditional materials, e.g., reinforced concrete. This study focuses on long one-dimensional prefabricated elements, which can be used for the construction of small self-supporting structures. During the development, emphasis was placed on the low weight of the elements and the simple way of joining them so that construction was possible without the use of mechanization. For long one-dimensional elements, which will be mainly subjected to loads induced by their own weight and environment-based loads such as from wind and snow, the prevalent type of load will be in the form of bending, while compressive loads will be minor. Hollow elements are a suitable solution for such conditions, having higher effective depth and elastic section modulus than solid elements of the same weight, and are used as transmission poles [1,2] and in marine structures [3,4]. The use of HPC ensures sufficient compressive strength, even with the reduced cross-section area [5]. The hollow core also provides space for installations and can be utilized for connecting individual prefabricated elements [1].

The flexural strength of hollow, traditionally reinforced concrete elements can be equal to the flexural strength of solid elements while having significantly lower weight [6,7,8,9]. The main limitations of the weakened hollow cross-section are lower shear strength which has to be considered in the design of the shear reinforcement, and also the compression zone, which will restrict the dimensions and position of the hollow core. Elements reinforced with fiber-reinforced polymer (FRP) bars have mostly similar reinforcement arrangements, with only steel stirrups being replaced with FRP spiral to provide shear resistance [10,11,12]. While thermoplastic resins allow the bending of the FRP bars to form stirrups [13], the FRP spirals are more commonly employed. Hollow columns with FRP spirals also showed higher ductility compared to hollow columns with FRP hoops/stirrups [14]. Along with the proposed woven FRP tubular mesh, the 3D printing of FRP reinforcement can supply other more advanced reinforcement structures [15]. The hollow core can be made either using removable formwork [6,7] or lost formwork, in which case the lost formwork can also be used for force transfer and complement the standard reinforcement [3,9]. However, it is not used as a sole reinforcement for load-bearing elements.

This study proposes hollow HPC beams with woven tri-axial carbon fiber composite reinforcement on the inner surface. The composite reinforcement made of carbon fibers is non-corrosive and does not require a concrete cover layer like traditional steel reinforcement [16,17]. The nominal concrete cover of traditional steel reinforcement can reach between 30 and 40 mm for structures in environment exposure class XC4 (alternately wet and dry) in accordance with Eurocode 2 [18], depending on the concrete class and allowance for deviation. The composite carbon reinforcement is very durable, and while the polymer matrix is to some extent negatively affected by the alkaline environment of the concrete matrix [19,20], it provides mechanical protection of the fine fibers and their homogenization. The concrete cover layer is relevant only in terms of reinforcement and concrete matrix interaction. However, sufficient cohesion can be achieved even with only one-sided contact of the reinforcement with the concrete matrix via surface modification of the composite reinforcement. The one-sided application of composite reinforcement is mainly used for the flexural [21,22,23] or shear [22,23,24,25] strengthening of existing concrete structures, but it can also be effectively applied as a surface reinforcement of planar elements [26].

Regarding the cohesion between reinforcement and concrete, the solution proposed by this study bears a stronger similarity to FRP composite bars. Despite its hollow core and the deviation of the fibers from the longitudinal axis of the composite reinforcement, the outer surface is still the contact zone between concrete and reinforcement and has to be adapted to provide proper interaction. The FRP bars are most commonly wounded, ribbed, or sand-coated to provide the required bond strength [27,28,29]. For the purposes of composite reinforcement without a solid core, the only applicable widely used solution is sand surface modification. This study also examines the possibility of using structural polyvinyl alcohol (PVA) fibers to improve this effect. Apart from enhancing cohesion, the reason for using PVA fibers is to achieve more ductile behavior of the hollow HPC elements with composite reinforcement [30,31] because the failure mode of the regularly used FRPs is very brittle. Applying the PVA fibers directly onto the surface of the composite reinforcement should also lead to a more advantageous load-parallel fiber orientation, similar to concrete elements with dispersed fibers longer than their thickness [32].

The main objective of this study was to evaluate the flexural performance of the novel woven CFRP tubular mesh positioned on the inner surface of the hollow HPC beams and the effect of the reinforcement surface modifications on its bond strength with the concrete matrix and on the ductility of the hollow beams. The study also investigated the utilization of the hollow core for the gluing of steel inserts as an option for connecting the elements. Lastly, the process of additive weaving of multiple layers of the composite reinforcement and its effect on the flexural strength of the elements was evaluated. The possible application of the lightweight and durable hollow HPC beams, which can be assembled without mechanization, was demonstrated via the construction of a small structure designed based on all the acquired data.

## 2. Materials and Methods

### 2.1. Materials

#### 2.1.1. High-Performance Concrete

The hollow concrete beams take advantage of the fact that the composite reinforcement requires only a minimal concrete cover layer. The concrete cover layer with a thickness of only a few millimeters requires the use of a high-performance fine-grained concrete mixture. The closed structure of the HPC matrix without capillary pores also provides excellent protection against the effects of chlorides, alkalis, and de-icing salts [33,34], which is essential for outdoor applications. The HPC mixture is given in Table 1. The compressive strength measured after 28 days on cubes with sides of 100 mm according to the technical standard EN 12390-3:2019 [35] was 138.2 ± 1.6 MPa. The tensile strength of concrete was measured on prisms with dimensions of 40 × 40 × 160 mm^3^ according to EN 12390-5:2019 [36] by three-point bending with a 100 mm support distance and an achieved average flexural strength of 8.54 ± 0.5 MPa. The static modulus of elasticity of HPC measured according to the technical standard ISO 1920-10:2010 [37] on prisms with dimensions 100 × 100 × 400 mm^3^ was equal to 49.1 ± 0.3 GPa. All tests were performed using the Controls MCC-Multitest testing machine (CONTROLS S.p.A., Milan, Italy).

#### 2.1.2. Composite Reinforcement

The composite reinforcement was prepared by the company Technofiber (TECHNOFIBER s.r.o., Slavkov u Brna, Czech Republic) using the weaving machine OS 144/1 (DOTEX, Nový Jičín, Czech Republic). The reinforcement was made of carbon rovings Tenax^®^-E STS40 F13 24K (Teijin Carbon Europe GmbH, Heinsberg, Germany) with a titer of 1600 tex. The basic parameters of the carbon roving were a tensile strength of 4300 MPa and a modulus of elasticity of 240 GPa, according to the technical data sheet [38]. The rovings were homogenized using the two-component epoxy resin SikaFloor-156^®^ (Sika Deutchland GmbH, Stuttgart, Germany) with a tensile strength in bending of 15 MPa and a modulus of elasticity of 2 GPa [39]. The mechanical performance of individually homogenized carbon rovings was evaluated. Tensile strength 3506 ± 233 MPa and the tensile modulus of elasticity 247 ± 12 GPa were measured. In order to compare the measured parameters with the parameters stated in the technical data sheets, only the theoretical cross-sectional area of the yarn itself was considered without the influence of the cross-sectional area of the epoxy resin, which only homogenized the material.

### 2.2. Specimen Preparation

All specimens consisted of an HPC shell, woven tri-axial composite reinforcement, and a hollow core. The woven reinforcement had carbon rovings orientated in two directions with a deviation of ±45° from the longitudinal axis. These rovings were woven on a template with the designed shape and dimensions, usually made of steel. The steel templates had rounded edges to prevent damage to the carbon fibers during weaving. Since the core fibrils of the carbon rovings would not be in direct contact with the concrete matrix, it would not be possible to achieve an effective engagement of all the fibrils, and the tensile strength of the reinforcement would be significantly lower without yarn homogenization [40]. Epoxy resin was therefore applied to homogenize the carbon fibrils. Before the curing of the epoxy resin, a surface treatment using fine-grained silica sand, PVA fibers, or their combination was applied.

**Figure 1:**
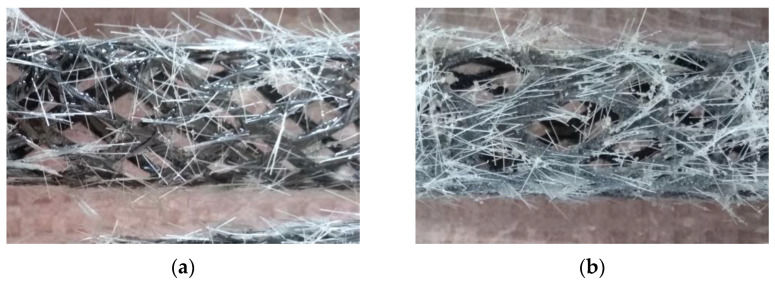
(**a**) Woven tri-axial composite reinforcement with a surface modified with MasterFiber 401 PVA fibers. (**b**) Woven tri-axial composite reinforcement with a surface modified with a combination of MasterFiber 401 PVA fibers and fine-grained silica sand.

The surface of the composite reinforcement was modified with different materials to improve the interaction of the composite reinforcement with the concrete matrix. Fine-grained silica sand ST 01/06 (Sklopísek Střeleč, a.s., Újezd pod Troskami, Czech Republic) with grain sizes from 0.1 mm to 0.6 mm and high SiO_2_ content of 99.2% [41] was used to improve the bond strength and prevent the delamination of the reinforcement from the concrete matrix. Structural PVA fibers MasterFiber 401 (Master Builders Solutions Deutschland GmbH, Trostberg, Germany) with a length of 12 mm were used to minimize the risk of shrinkage crack development and to prevent fragments of the concrete matrix of damaged elements from spalling. The fine-grained silica sand, PVA fiber (Figure 1a), or both (Figure 1b) were poured over freshly impregnated carbon rovings and were embedded in the epoxy resin before curing. The method of application led to variations in the amount of material that merged with the composite reinforcement. Therefore, all values indicating the weight of the applied material are only approximate.

**Figure 2:**
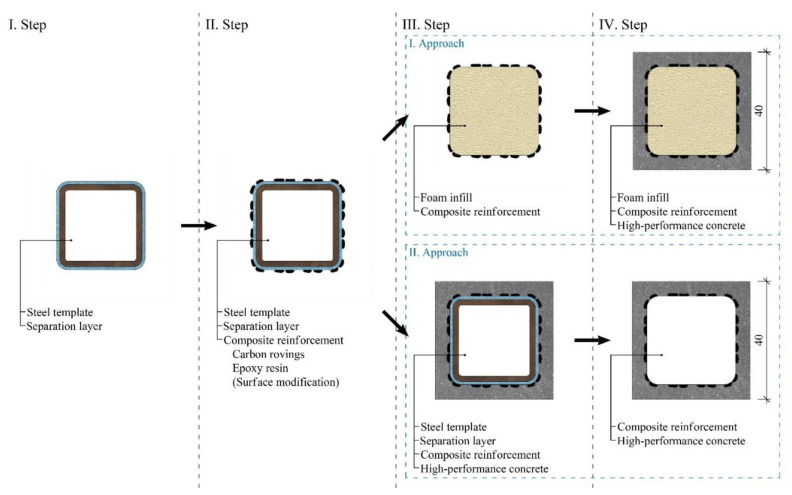
Manufacturing process of the hollow HPC beams with one layer of the woven composite reinforcement.

The manufacturing procedure of the hollow HPC beams is described in Figure 2. The first step involves the preparation of steel templates for weaving the composite reinforcement and covering them with a separation layer for easy removal of the template. The second step comprises the weaving of the carbon rovings, the impregnation with epoxy resin, and surface modifications. These composite reinforcements were shortened to a length of 1200 mm. The composite reinforcement had to be placed into a mold before the casting of the concrete, and two different approaches were used to ensure its correct position. The first approach presented in the upper half of Figure 2 was to remove the steel template used for weaving the reinforcement and filling the resulting cavity with expanding foam to prevent the concrete from penetrating into the core during casting, which was the third step. The structural stiffness of the composite reinforcement was sufficient for the distance of 1200 mm to be fixed only on the ends without any additional spacers. The preparation was completed in the fourth step with the casting and de-molding of the concrete.

The second approach presented at the bottom half of Figure 2 was to use the steel template for reinforcement positioning. The steel template was covered by a thin foam separation layer to avoid problems during the removal of the template caused by the shrinkage of the HPC. This separation layer had to be resistant to solvents because of the application process of epoxy resin. The third step, therefore, comprised placing the composite reinforcement into a mold and the casting of concrete. The steel template was removed after the hardening of the concrete in step four.

**Figure 3:**
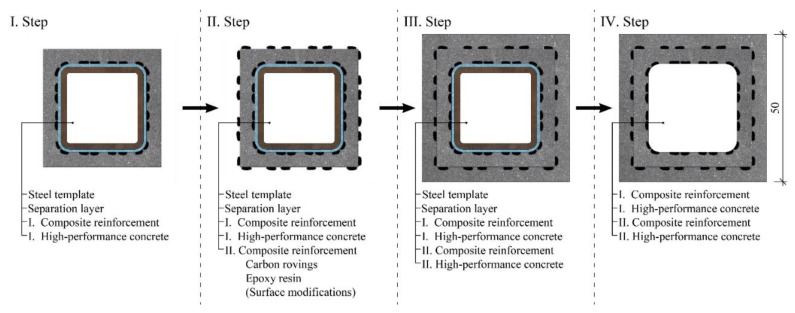
Manufacturing process of the hollow HPC beams with two layers of the woven composite reinforcement.

The possibility of utilizing multiple layers of woven composite reinforcement was also explored. In this case, previously prepared hollow concrete beams were used in place of the steel template for weaving a second layer of the composite reinforcement. No separation layer was used to ensure cohesion of the second layer of reinforcement with the inner concrete shell. Epoxy resin impregnation and surface modification using fine-grained silica sand, PVA fibers, or both were applied similarly to the production of the composite reinforcement on the steel template, as illustrated in the second step in Figure 3. These concrete shells with composite reinforcement on both the inner and outer surface were then placed into a mold for the casting of the concrete. The steel template used for the weaving of the initial layer of the composite reinforcement was used for the positioning of the reinforcement. The last step consisted of the removal of the steel template and the de-molding.

### 2.3. Testing Methods

It was assumed that the hollow beams would be subjected to stresses mainly induced by bending resulting from their own weight or from other minor loads. Bending stress is one of the best-described behaviors of reinforced and textile-reinforced concrete in real conditions. The testing can be divided into three parts, a four-point bending test to evaluate the effect of different surface modifications of the composite reinforcement, the testing of the developed glued joints of the hollow concrete beams, and a three-point bending test to evaluate the effect of multiple layers of the composite reinforcement. The three-point and four-point bending tests were carried out according to the technical standard EN 12390-5:2019 [36]. All testing was performed using the LabTest 4.100SP1 testing machine (LaborTech Ltd., Opava, Czech Republic).

#### 2.3.1. Composite Reinforcement Surface Modifications

The influence of the surface modifications on the interaction of the inner composite reinforcement with the concrete shell was evaluated using a four-point bending test. The distance between support pins was 300 mm and 100 mm between loading pins, and the testing was performed with a constant increment of displacement of 5 mm/min. Five sets of hollow beam specimens were prepared with a 40 × 40 mm^2^ cross-section, a length of 360 mm, and composite reinforcement woven from 24 carbon rovings. Specimens with composite reinforcement with an unmodified surface and composite reinforcement with a surface modified with ~50 g/m^−^ and ~100 g/m^−^ of fine-grained silica sand had a hollow core with a cross-section of 28 × 28 mm^2^. Specimens with composite reinforcement surface modified with ~5 g/m^−^ of PVA fibers and a combination of ~5 g/m^−^ of PVA fibers and ~50 g/m^−^ of fine-grained silica sand had a hollow core with a cross-section of 30 × 30 mm^2^. The difference in the hollow core dimensions was a result of different approaches during specimen preparation.

#### 2.3.2. Glued Joints Testing

**Figure 4:**
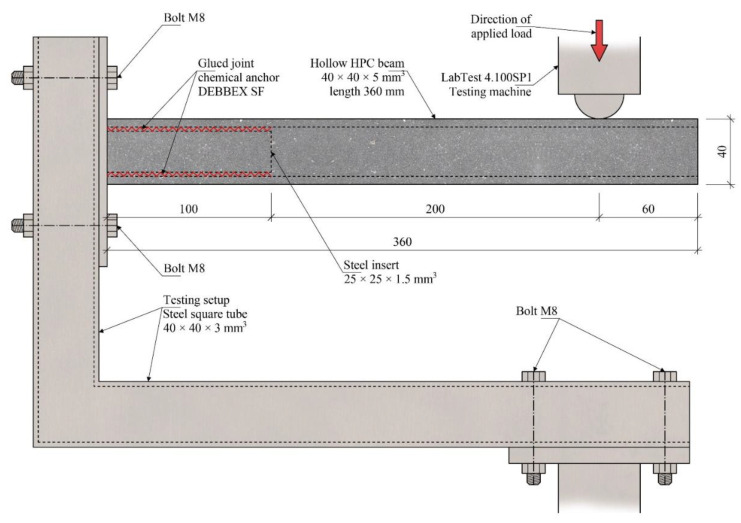
Glued joints testing setup.

A solution for connecting the hollow beams was designed via gluing steel parts into the hollow core: either gluing end elements designed for articulated connection to steel structures or gluing elements designed for rigid connection of the hollow beams to each other, for example, in the corners of the structure. A testing setup shown in Figure 4 was designed to evaluate the interaction of the hollow beams and the glued steel insert. Testing was performed with a constant increment of displacement of 5 mm/min at the distance of 200 mm from the end of the steel insert. The tested hollow beam specimens had dimensions of 40 × 40 × 360 mm^3^ with a hollow core of 30 × 30 mm^2^ and were reinforced with a composite reinforcement with a surface modified with ~5 g/m^−^ of PVA fibers.

**Figure 5:**
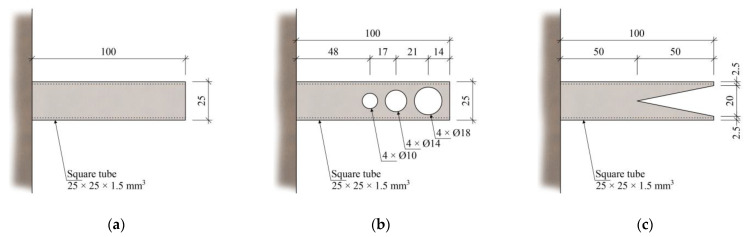
(**a**) Steel insert with constant stiffness; (**b**) perforated steel insert; and (**c**) cutout steel insert.

Three steel inserts with varying degrees of stiffness were designed to better investigate the behavior of the glued joint and the development of cracks around the end of the steel insert. The inserts with a cross-section of 25 × 25 mm^2^ and a length of 100 mm were designed to fit the specimen hollow cores. Figure 5a shows an insert with constant stiffness, while Figure 5b,c shows inserts with decreasing stiffness towards their ends. The inserts were glued inside the hollow core using chemical anchor DEBBEX SF polyester (Den Braven Czech and Slovak, a.s., Úvalno, Czech Republic).

#### 2.3.3. Multi-Layer Woven Composite Reinforcement

A three-point bending test was used to investigate the basic load-bearing characteristics of the hollow beams with multi-layer composite reinforcement. The distance between support pins was 120 mm, and the testing was performed with a constant increment of displacement of 2 mm/min. Four sets of hollow beam specimens were prepared with a 50 × 50 mm^2^ cross-section and a length of 160 mm. In this case, the hollow core had a cross-section of only 26 × 26 mm^2^. The composite reinforcement was woven in variants with 24 and 36 carbon rovings in both the inner and outer layers. Both sets were complemented with reference to only the inner layer of the composite reinforcement with a corresponding number of carbon rovings.

## 3. Results and Discussion

### 3.1. Composite Reinforcement Surface Modifications

**Figure 6:**
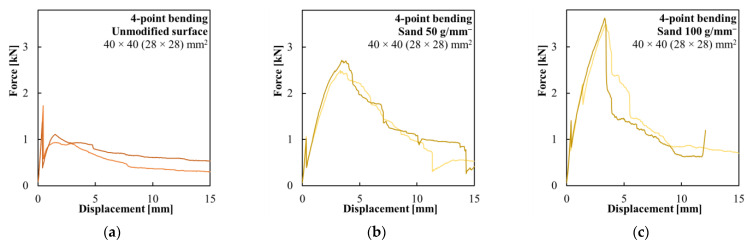
Force–displacement diagrams of the four-point bending test of the hollow beams with (**a**) composite reinforcement without surface modification; (**b**) composite reinforcement modified with 50 g/m^−^ of fine-grained silica sand; and (**c**) composite reinforcement modified with 100 g/m^−^ of fine-grained silica sand.

The first set of specimens was tested to determine the impact of fine-grained silica sand and structural PVA fibers on the cohesion of the composite reinforcement and the concrete matrix. Figure 6a shows the results of the specimens with the composite reinforcement with an unmodified surface. Stress release during the development of the first crack led to the delamination of the smooth composite reinforcement from the concrete shell, and the reinforcement provided only minimal residual flexural strength after the crack developed. Figure 6b shows the results of the specimens with the composite reinforcement with a surface modified with 50 g/m^−^ of fine-grained silica sand. There was a significant increase in the transmitted force after the development of the first crack before the reinforcement gradually delaminated from the concrete shell. Figure 6c shows the results of the specimens with the composite reinforcement with a surface modified with 100 g/m^−^ of silica sand. Those specimens achieved the highest ultimate flexural strength and had very brittle behavior.

The surface modifications generally led to a weakening of the thin concrete shell, signified by earlier crack development. The fine-grained silica sand applied on the composite reinforcement surface improved cohesion with the concrete shell. A higher amount of used silica sand correlated with higher flexural strength and more brittle behavior. The composite reinforcement modified with a lower amount of silica sand lost cohesion with the concrete shell more gradually, while the composite reinforcement with a higher amount of silica sand collapsed suddenly without any prior warning. In all cases, the failure of the specimen was caused by the delamination of the composite reinforcement.

**Figure 7:**
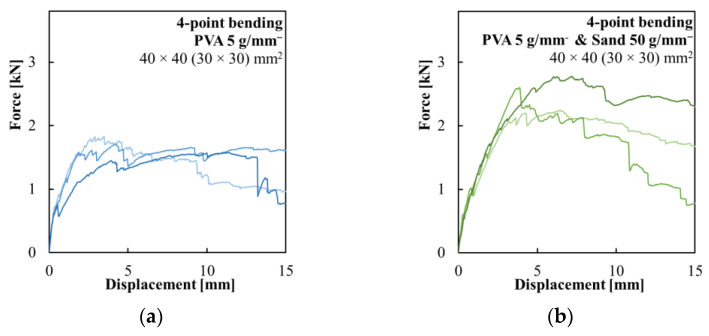
Force–displacement diagrams of the four-point bending test of the hollow beams with (**a**) composite reinforcement modified with 5 g/m^−^ of PVA fibers and (**b**) composite reinforcement modified with a combination of 5 g/m^−^ of PVA fibers and 50 g/m^−^ of fine-grained silica sand.

The results of specimens with PVA fibers used to modify the surface of the composite reinforcement are presented in Figure 7a. The use of structural PVA fibers improved the cohesion of the reinforcement with the concrete shell. The ultimate flexural strength was lower compared to the hollow beams with silica sand modified composite reinforcement, but their behavior was significantly more ductile. Figure 7b shows the results of specimens with the reinforcement surface modified with a combination of PVA fibers and fine-grained silica sand. These specimens had ductile behavior similar to the specimens coated only with PVA fibers, but achieved higher flexural strength. The ultimate flexural strength was lower compared to the hollow beams with sand-coated composite reinforcement. The use of PVA fibers led to a lower stiffness before crack initiation compared to specimens with only silica sand surface modification but also prevented the spalling of concrete fragments despite a significant deflection of the specimens during the testing.

Due to the variance in the dimensions of the specimens, there are differences in their elastic section moduli and effective depth of cross-section. To complement the force/displacement diagrams, the elastic section moduli, first-cracking and ultimate loads, and calculated first-cracking stress are summarized in Table 2, and all parameters are compared relative to the composite reinforcement with an unmodified surface.

### 3.2. Glued Joints Testing

**Figure 8:**
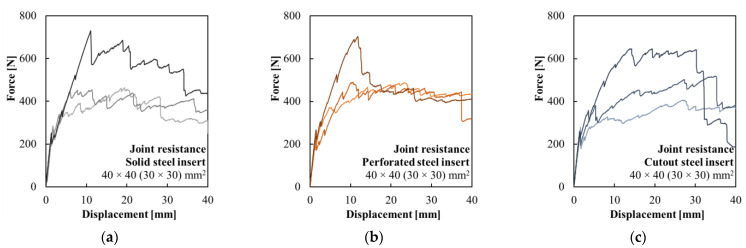
Force–displacement diagrams of the testing of hollow HPC beams with a glued: (**a**) steel insert with constant stiffness; (**b**) perforated steel insert; and (**c**) cutout steel insert.

The testing of specimens with a glued steel insert with constant stiffness is presented in Figure 8a. All specimens show the engagement of the composite reinforcement after the development of the first crack in the concrete shell. Similar results were achieved by specimens with glued perforated steel inserts (Figure 8b) and specimens with glued cutout steel inserts (Figure 8c). The composite reinforcement with a PVA-fiber-modified surface ensured the predictable ductile behavior of the elements with multi-crack development along the length of the element. While cracks formed in the concrete shell around the steel insert, there was no sign of the delamination of the chemical anchor glued joint. The mode of failure of all specimens was the delamination of the composite reinforcement from the concrete shell.

The testing of all variants yielded very similar results, as given in Table 3. However, the lower rigidity of the steel insert led to a higher calculated stress in the concrete shell at the initiation of the crack development. Considering the ratio between the elastic section moduli, this increase was equivalent to 3% in the case of the perforated steel insert and 11% for the cutout steel insert compared to the solid steel insert. The average ultimate loading force of all variants differed only slightly, but due to the mode of failure, the standard deviation was high.

### 3.3. Multi-Layer Woven Composite Reinforcement

**Figure 9:**
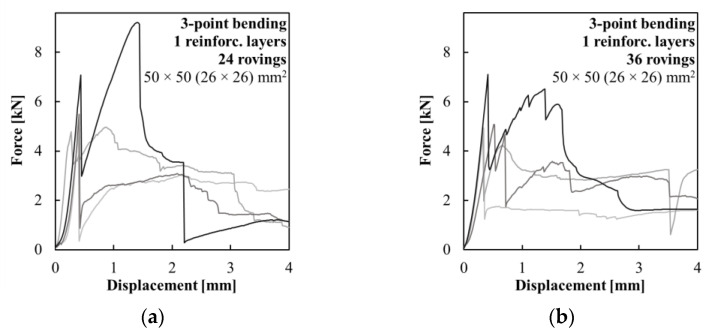
Force–displacement diagrams of the three-point bending test of the hollow beams with one layer of composite reinforcement woven from (**a**) 24 carbon rovings and (**b**) 36 carbon rovings.

The last experiment focused on the mechanical performance of the hollow HPC beams with multi-layer composite reinforcement. Figure 9 shows a comparison of specimens with one-layer composite reinforcement woven from 24 (Figure 9a) and 36 (Figure 9b) carbon rovings. Those specimens served as a reference for those with two layers of composite reinforcement. There was a high variance of results in the control series of specimens with both 24 and 36 roving composite reinforcement. This issue was a result of the position of the reinforcement, which was covered by a stronger concrete shell of approximately 11 mm. This, after crack development in the concrete shell, most often led to the delamination of a large surface area of the composite reinforcement, which then provided only low residual flexural strength to those elements. The average measured ultimate loading force was only 10% (24 roving variant) and 5% (36 roving variant) higher than the force at the time of crack development.

**Figure 10:**
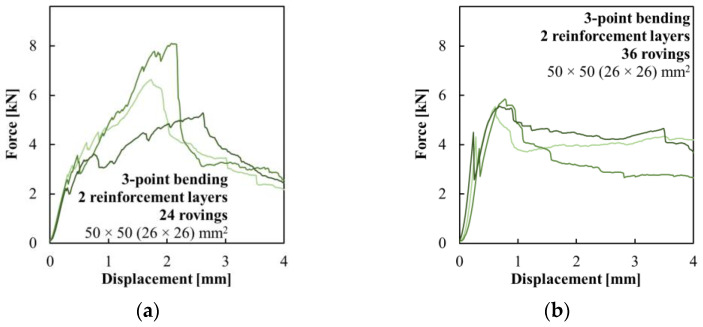
Force–displacement diagrams of the three-point bending test of the hollow beams with two layers of composite reinforcement woven from (**a**) 24 carbon rovings and (**b**) 36 carbon rovings.

The results in Figure 10 demonstrate that the second layer of composite reinforcement provided insufficient interaction between the inner and outer concrete shells, which led to the early development of a crack. The variant with 24 rovings woven reinforcement achieved only 47% of the first-crack stress compared to the one-layer composite reinforcement variant. The specimens with 36 roving reinforcement performed better but still reached only 76% of the reference value. The composite reinforcement was effectively engaged after the development of the crack, but the average ultimate flexural strength of the specimens with two-layer composite reinforcement was only 8% higher than of those with one-layer reinforcement in the case of the reinforcement woven from 24 rovings, while it was even lower, 1%, in the case of the 36 rovings variant, as given in Table 4. The main issue was caused by the epoxy resin impregnation. The composite reinforcement prepared on the steel template had openings between the carbon rovings after the removal of the template. When the hollow concrete beam was used as a template for weaving a second layer of the composite reinforcement, the epoxy resin formed a continuous layer around the concrete element. Since the high-performance concrete used in this experiment had a closed structure, and the surface modification could be applied only on the outside of the second layer of the composite reinforcement, the cohesion between those two was very limited.

## 4. Application

**Figure 11:**
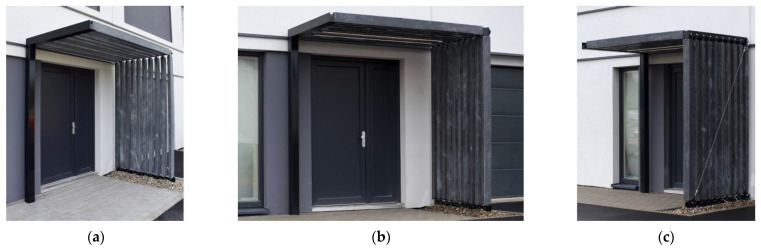
Demonstration of a possible application in the form of a small shading structure—a pergola: (**a**) detail of the steel support structure; (**b**) frontal view of the pergola; and (**c**) detail of wind bracing.

After the viability of the developed technology was assessed, a small structure made of the hollow HPC beams with inner composite reinforcement was constructed to illustrate the possibilities of the developed technology. The structure shown in Figure 11 is a pergola—a shading structure that outlines the area around the entrance of an experimental building on the premises of the University Centre for Energy Efficient Buildings of CTU in Prague (UCEEB CTU in Prague, Buštěhrad, Czech Republic).

The structure was designed as a combination of load-bearing steel construction, steel glued joints, and hollow HPC elements. The structure was designed from hollow beams and columns of a rectangular cross-section with dimensions of 60 × 120 mm^2^. The hollow core had a cross-section of 44 × 104 mm^2^, leaving the HPC concrete shell with a thickness of approximately 8 mm. The composite reinforcement was woven from 24 carbon rovings (1600 tex). Its surface was modified with a combination of PVA fibers and fine-grained silica sand, which achieved both high flexural strength and ductility during testing. The length of both horizontal and vertical elements was 2340 mm.

Due to the lightweight elements, the pergola could be assembled without the use of heavy handling equipment. The stability of the pergola was ensured via wind bracing using a stainless-steel wire with a diameter of 5 mm in the vertical plane. The pergola was also equipped with LED lights embedded directly in two of the horizontal hollow beams to provide illumination. The power supply to the LED light was designed to pass through the hollow core of both horizontal and vertical elements.

## 5. Conclusions

This study examined and discussed the mechanical performance of the hollow HPC beams with composite reinforcement on their inner surface. The effect of surface modifications of the composite reinforcement was assessed, alongside testing of the connections of the hollow elements and the use of multiple layers of the woven composite reinforcement. The main conclusions of this study are summarized as follows:

- The proposed surface modifications of the inner woven composite reinforcement proved to have a significant effect on the bending behavior of the hollow beams. The composite reinforcement, without any surface modification, proved unsuitable for any application as the unmodified reinforcement delaminated from the HPC shell after crack development. Coating the surface with either fine-grained silica sand or PVA fibers improved bond strength with the cementitious matrix. The sand coating led to higher flexural strength and more brittle behavior, while the elements with the PVA fibers had lower flexural strength and were more ductile. The use of both surface treatments together resulted in an optimal combination of flexural strength and ductility suitable for the proposed long one-dimensional prefabricated elements.
- Experimental loading showed that the glued joints of the hollow HPC beams using a chemical anchor provided a viable solution for connecting them and equipping them with end elements. The glued connection was reliable, and all specimens failed due to the delamination of the composite reinforcement from the inner surface of the hollow HPC beam. The stiffness of the glued steel insert affected the crack development initiation. The lower stiffness of the insert led to higher flexural strength at first crack development.
- The hollow HPC elements reinforced with the multi-layer woven composite reinforcement proved not to be viable with the proposed technology. The additive production technology of weaving carbon rovings directly around previously cast HPC elements, and especially the subsequent impregnation of the rovings using epoxy resin, led to the insufficient interaction of the inner and outer concrete shells, which led to the delamination and early failure of the tested specimens.
- The construction of the small self-supporting structure designed based on the findings of this study demonstrated the feasibility of assembling the hollow concrete elements without the use of mechanization. The use of a chemical anchor for gluing steel joints was proven fast and reliable. The hollow core was also used for the concealed wiring of the LED lights built into the surface of the concrete beams.

The presented woven composite reinforcement on the inner surface of the hollow HPC elements had rovings orientated only in two directions, orientated at ±45° from the longitudinal axis. It would also be possible to apply additional rovings in the longitudinal direction. The bending stiffness of such elements would increase significantly. However, no longitudinally oriented rovings were needed for elements designed for the self-supporting structure presented in this study. Further research will focus on the development of 1D elements (columns and beams) with the woven composite reinforcement in combination with longitudinal reinforcement and their use as load-bearing structures.

## 6. Patents

Patent “Lightweight concrete element with textile reinforcement and a method of its production” licensed to CTU in Prague and TECHNOFIBER, s. r. o.

## Figures and Tables

**Table 1:** High-performance concrete mix composition.

Component	[kg/m^3^]
Cement I 42.5 R	680
Technical silica sand	960
Silica flour	325
Silica fume	175
Superplasticizers	29
Water	171

**Table 2:** Summary of measured and calculated characteristics of specimens with and without composite reinforcement surface modifications.

Series	Amt.	Elastic Section Modulus [mm^3^]	First-Cracking Load [N]	First-Cracking Stress [MPa]	Ultimate Loading Force [N]
Unmodified surface	2	8932 ± 163 *(100%)*	1557.6 ± 174.5 *(100%)*	8.70 ± 0.82 *(100%)*	1557.6 ± 174.5 *(100%)*
Silica sand 50 g/m^−^	2	9239 ± 18 *(103%)*	1046.1 ± 27.7 *(67%)*	5.66 ± 0.14 *(65%)*	2607.0 ± 114.2 *(167%)*
Silica sand 100 g/m^−^	2	9070 ± 277 *(102%)*	1338.2 ± 68.1 *(86%)*	7.39 ± 0.60 *(85%)*	3555.7 ± 66.6 *(228%)*
PVA fibers 5 g/m^−^	3	9133 ± 441 *(102%)*	689.3 ± 45.5 *(44%)*	3.78 ± 0.23 *(43%)*	1705.4 ± 104.3 *(110%)*
PVA fibers + Silica sand	3	8777 ± 329 *(98%)*	612.8 ± 45.5 *(39%)*	3.49 ± 0.23 *(40%)*	2549.5 ± 225.2 *(164%)*

**Table 3:** Summary of measured and calculated characteristics of specimens with steel inserts glued inside their hollow cores.

Series	Amt.	Elastic Section Modulus [mm^3^]	First-Cracking Load [N]	First-Cracking Stress [MPa]	Ultimate Loading Force [N]
Solid steel insert	3	8712 ± 187 *(100%)*	256.1 ± 21.7 *(100%)*	6.02 ± 0.38 *(100%)*	550.0 ± 128.1 *(100%)*
Perforated steel insert	3	8122 ± 504 *(93%)*	229.3 ± 26.8 *(90%)*	5.79 ± 0.58 *(96%)*	560.5 ± 101.9 *(102%)*
Cutout steel insert	3	8397 ± 98 *(96%)*	264.8 ± 10.9 *(103%)*	6.46 ± 0.28 *(107%)*	524.0 ± 97.7 *(95%)*

**Table 4:** Summary of measured and calculated characteristics of specimens with one and two layers of the composite reinforcement.

Series	Amt.	Elastic Section Modulus [mm^3^]	First-Cracking Load [N]	First-Cracking Stress [MPa]	Ultimate Loading Force [N]
One layer 24 rovings	4	21,699 ± 759 *(100%)*	5613.6 ± 887.1 *(100%)*	7.75 ± 1.09 *(100%)*	6189.4 ± 1754.1 *(100%)*
Two layers 24 rovings	3	21,726 ± 118 *(100%)*	2648.3 ± 288.5 *(47%)*	3.65 ± 0.38 *(47%)*	6674.3 ± 1156.9 *(108%)*
One layer 36 rovings	4	20,763 ± 930 *(100%)*	5440.6 ± 940.9 *(100%)*	7.87 ± 1.38 *(100%)*	5702.3 ± 1202.7 *(100%)*
Two layers 36 rovings	3	21,271 ± 1121 *(102%)*	4224.8 ± 273.7 *(78%)*	5.99 ± 0.66 *(76%)*	5652.1 ± 141.1 *(99%)*

## Data Availability

The data presented in this study are available on request from the corresponding author. The data are not publicly available.
